# Ionic Liquids as Working Fluids for Heat Storage Applications: Decomposition Behavior of N-Butyl-N-methylpyrrolidinium tris(pentafluoroethyl)trifluorophosphate

**DOI:** 10.3390/ma16051762

**Published:** 2023-02-21

**Authors:** Francesca Nardelli, Enrico Berretti, Alessandro Lavacchi, Emanuela Pitzalis, Angelo Freni, Silvia Pizzanelli

**Affiliations:** 1 Italian National Council for Research—Institute for the Chemistry of OrganoMetallic Compounds, CNR-ICCOM, Via G. Moruzzi 1, 56124 Pisa, Italy; 2 Italian National Council for Research—Institute for the Chemistry of OrganoMetallic Compounds, CNR-ICCOM, Via Madonna del Piano 10, 50019 Firenze, Italy; 3 Centre for Instrument Sharing (CISUP), University of Pisa, Lungarno Pacinotti 43, 56126 Pisa, Italy

**Keywords:** ionic liquids, thermal degradation, metal corrosion, N-butyl-N-methylpyrrolidinium tris(pentafluoroethyl)trifluorophosphate, [BmPyrr]FAP, HRMAS NMR, ICP-OES, EDX

## Abstract

Ionic liquids (ILs) represent promising working fluids to be used in thermal energy storage (TES) technologies thanks to their peculiar properties, such as low volatility, high chemical stability, and high heat capacity. Here, we studied the thermal stability of the IL N-butyl-N-methylpyrrolidinium tris(pentafluoroethyl)trifluorophosphate ([BmPyrr]FAP), a potential working fluid for TES applications. The IL was heated at 200 °C for up to 168 h either in the absence or in contact with steel, copper, and brass plates to simulate the conditions used in TES plants. High-resolution magic angle spinning nuclear magnetic resonance spectroscopy was found to be useful for the identification of the degradation products of both the cation and the anion, thanks to the acquisition of ^1^H, ^13^C, ^31^P, and ^19^F-based experiments. In addition, elemental analysis was performed on the thermally degraded samples by inductively coupled plasma optical emission spectroscopy and energy dispersive X-ray spectroscopy. Our analysis shows a significant degradation of the FAP anion upon heating for more than 4 h, even in the absence of the metal/alloy plates; on the other hand, the [BmPyrr] cation displays a remarkable stability also when heated in contact with steel and brass.

## 1. Introduction

Development of new generation thermal energy storage (TES) technologies is a factor of primary importance for efficient use of solar thermal energy as well as industrial waste or process heat [1]. TES efficiency noticeably depends on the thermo-physical properties of the working heat storage material, which has to offer high performance in terms of storage capacity and stability under required operating conditions [2]. In case of solar concentrating power plants, water, diathermic oils, and molten salts are used as conventional working fluids [3]. Phase change materials were also proposed in order to increase the heat storage density [4]. However, these materials suffer from several limitations, such as high vapor pressure, high melting point, low thermal conductivity, in addition to an electrochemical behavior possibly leading to corrosion of metallic plant materials [5].

Ionic liquids (ILs) were suggested as efficient and sustainable working fluids in solar energy technologies thanks to their unique properties, such as low vapor pressure, high heat capacity, low melting point, and relatively high density [6,7]. Additionally, ILs possess high chemical stability, very low flammability and volatility, with subsequent low impact on the environment and health [8,9], and are relatively easy to tune for specific purposes just by changing the cation/anion pair or by modifying their functional groups [10].

Notwithstanding the noticeable number of studies focused on the application of ILs as heat transfer fluids and thermal storage media [9,11], far fewer are the studies that investigate ILs stability under real operating conditions, which is an important prerequisite for their actual implementation in TES systems. In fact, several factors may play a role in ILs degradation, including the applied temperature and time, the presence of impurities, and the contact with air and with metals/alloys constituting thermal circuits of the plants.

Most of the thermal stability studies available in the literature are based on thermogravimetric analyses (TGA) [12,13,14]. However, the heating time used in specific TGA measurements trying to simulate applicative conditions, such as long-term isothermal experiments, is still far lower than that employed in real processes. Furthermore, this methodology does not allow the identification of the degradation species. To this aim, several techniques coupled to mass spectrometry were applied, including gas chromatography, pyrolysis, TGA, thermal desorption, ion chromatography, or capillary electrophoresis [12,15,16,17]. Sometimes, thermal stability of ILs was investigated by ultraviolet, infrared, X-ray photoelectron, or nuclear magnetic resonance (NMR) spectroscopies [16]. In particular, NMR spectroscopy is especially powerful because it can detect different types of nuclei, the most common being ^1^H, ^13^C, ^19^F, and ^31^P, and allows a plethora of experiments to be acquired, thus partially overcoming the problem of signal overlap [18,19]. Specifically, high-resolution magic angle spinning (HRMAS) NMR is useful to characterize materials exhibiting a high viscosity, such as ILs; in fact, this NMR technique relies on spinning the sample at high speeds at the “magic” angle, allowing to completely cancel out the residual anisotropic interactions and obtain highly resolved spectra, which cannot be achieved using solution state NMR [20,21]. Indeed, HRMAS NMR proved to be useful to identify the degradation products of N-tributyl-N-methylammonium bis(trifluoromethanesulfonyl)imide [22].

In this work, we took advantage of HRMAS NMR to characterize the thermal degradation of 1-butyl-1-methylpyrrolidinium tris(pentafluoroethyl)trifluorophosphate, [BmPyrr]FAP. This IL was selected for potential employment as heat transfer fluid thanks to the high thermal stability of the cation and anion, as reported in the literature. In fact, some studies showed that [BmPyrr] cation is rather stable if it is heated at 60 or 95 °C for up to 16 months [16,17]. On the other hand, the FAP anion was introduced to overcome the limited thermal and hydrolytic stability of hexafluorophosphate anion ([PF_6_]^−^), thanks to the presence of hydrophobic perfluoroalkyl substituents [23,24]. Here, [BmPyrr]FAP was heated at 200 °C for 168 h and monitored at the beginning, at two intermediate times and at the end of the thermal treatment. We also investigated the thermal degradation of [BmPyrr]FAP in the presence of three different metal/alloy plates (steel, brass, and copper) to simulate the contact of the ionic liquid with metals/alloys as it happens in power plants. Corrosion phenomena of different metals/alloys in contact with other ILs were reported and ascribed to the oxidation of metals by dissolved oxygen [25,26]. Our purpose was to reveal whether the metal/alloy affects the degradation of [BmPyrr]FAP. The content of metals dissolved in the IL was detected via inductively coupled plasma optical emission spectroscopy (ICP-OES). Moreover, the relative concentration of the elements constituting the investigated ionic liquid (C, N, O, F, and P) in the bulk of the most aged samples was estimated by energy dispersive X-ray (EDX) microanalysis.

## 2. Materials and Methods

### 2.1. Materials

**Scheme 1:**
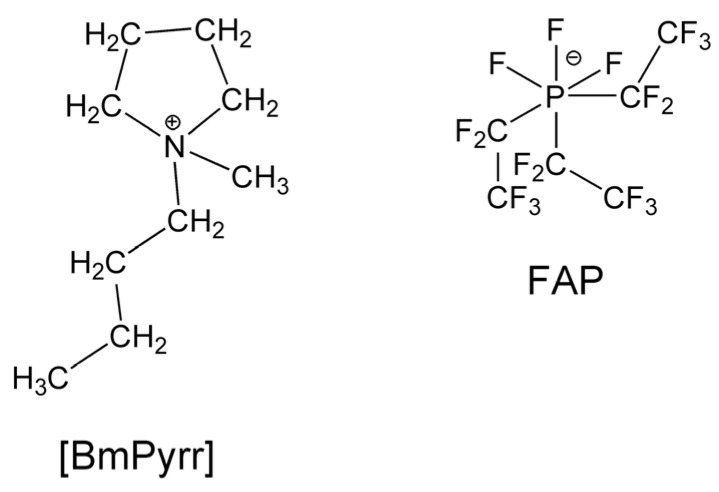
Chemical structure of the [BmPyrr] cation and the FAP anion of the studied IL.

The ionic liquid [BmPyrr]FAP (Scheme 1), MW = 587.27 g/mol, was purchased from Merck KGaA (purity: 99.0%; halides ≤ 100 ppm; water ≤ 100 ppm). The presence of water in the commercial product was also checked using HRMAS NMR. The ^1^H spectrum recorded on the pure IL, without the addition of any solvent, did not show any water signal, indicating that the water content is below the NMR detection limit (data not shown).

The ionic liquid was heated in an oven at 200 °C for 168 h in the presence of a steel, copper, or brass metal plate (2 cm × 2 cm × 0.07 cm) or with no metal plate. We used AISI 31XX series steel (wt%: Ni < 1.25, Cr < 0.60), commercial copper (purity > 99.9%), and rivet brass (wt%: Cu 63, Zn 37). In order to check the nominal amounts, EDX analyses were performed on the metal plates prior to the immersion into the IL, confirming the expected values, except for steel, where only Fe was detected, because Ni and Cr contents are below the detection limit. The metals/alloys used in this work were kindly provided by a company in Arezzo (Italy). At intermediate heating times (4 and 24 h), 1 mL of IL was sampled for NMR spectroscopy and ICP-OES analyses.

Table 1 summarizes the codes of the analyzed samples used throughout the text. The code of the initial non-heated sample is blank_0h.

### 2.2. HRMAS NMR

NMR spectra were acquired on a Bruker AVANCE NEO NMR spectrometer, working at the Larmor frequencies of 500.13, 125.77, 202.45, and 470.56 MHz for ^1^H, ^13^C, ^31^P, and ^19^F nuclei. A HRMAS 4 mm probe was used for ^1^H, ^13^C, and ^31^P-based experiments, whereas a 4 mm cross-polarization MAS probe was used for ^19^F measurements. DMSO-d_6_ (99.7% deuterated, Sigma) was added to the samples in order to, at least partially, dissolve them and also for deuterium locking purposes. HRMAS 4 mm rotors with a 50 μL volume were used. For liquid samples, solutions with DMSO-d_6_ were prepared (1:1 volume ratio), and then 50 μL of each mixture was transferred to the rotor; in the case of solid or semi-solid samples, they were directly loaded into the rotor, in order to fill half of the rotor volume, and then added with about 20–30 μL DMSO-d_6_. Tetramethylsilane was added for ^1^H and ^13^C internal spectral referencing. For ^31^P and ^19^F experiments, the chemical shift (δ) scale was externally calibrated using CFCl_3_ (δ(^19^F): 0 ppm) and 85% H_3_PO_4_ (δ(^31^P): 0 ppm).

For selected samples, ^1^H, ^13^C, ^31^P, and ^19^F spectra were acquired. For ^1^H, ^13^C, ^31^P, and ^19^F nuclei excitation, 90° pulses of 7, 12, 12, and 5 μs were used, respectively. ^1^H spectra were acquired using the one pulse experiment, with a relaxation delay of 1–2 s and accumulating 64–1024 scans. ^13^C spectra were recorded for samples blank_0h, brass_168h, and blank_168h only, using the Bruker zg30pg pulse sequence for NOE enhancement of carbon nuclei signals, using a relaxation delay of 2 s and accumulating 4k scans. ^31^P spectra were recorded on samples blank_0h, blank_4h, blank_24h, blank_168h, steel_4h, steel_24h, steel_168h, brass_168h, and copper_168h only, using the one pulse experiment. A relaxation delay of 6 s was applied. ^19^F spectra were recorded on samples blank_0h and blank_168h only, using the one pulse experiment with a relaxation delay of 3 s. The assignment of the signals in the ^1^H spectrum of blank_0h was supported by ^1^H-^13^C heteronuclear single quantum coherence (HSQC) correlation experiment. One-dimensional (1D) selective ^1^H total correlation spectroscopy (TOCSY) and HSQC experiments were also recorded on blank_168h for signal assignment of the cation degradation products. The HSQC experiment was also performed on brass_168h. The Bruker seldigpzs pulse sequence was used for the acquisition of ^1^H 1D-TOCSY experiments, applying a Gaussian-shaped 180° pulse for selective excitation. The relaxation delay was set to 1 s, and the mixing time to 80 ms. For the ^1^H-^13^C HSQC experiments, the Bruker hsqcetgpsisp2.2 pulse sequence was employed, with a relaxation delay of 1–1.5 s. All measurements were recorded using a MAS spinning rate of 6 kHz and maintaining the temperature at 298 K.

### 2.3. ICP-OES Measurements

The amount of the metals in the samples was quantified by ICP-OES. The mineralization was carried out with a Milestone Start D microwave digestor (FKV S.r.l., Torre Boldone, Italy). Weighed portions of approximately 100 mg of sample were treated with 6 mL HNO_3_ (Fluka, TraceSELECT^TM^ for trace analysis, >69%) and 2 mL H_2_O_2_ (Supelco, Suprapur^®^, 30%). After the thermal cycle in the closed vessels, the solutions were properly diluted with ultrapure water (18.2 MΩ·cm, PureLab Pro, ELGA LabWater, High Wycombe, Bucks, UK) for ICP-OES analysis. Calibration curves were obtained by dilution of commercial standard solutions (Fluka, TraceCERT©) in HNO_3_ 2%. ICP-OES measurements were carried out with an Optima 8000 ICP-OES (Perkin Elmer, Waltham, MA, USA) operating at 1500 W and equipped with an autosampler S10, MiraMist^®^ Nebulizer (Perkin Elmer, Waltham, MA, USA) and cyclonic chamber. Argon (420.069 nm) was used as the internal standard. Each element was measured at the following wavelengths (nm): Fe (238.204), Ni (231.604), Cr (267.716), Cu (327.393), Zn (206.200), and Sn (189.927).

### 2.4. EDX Measurements

EDX acquisition was performed using an EDAX OCTANE detector mounted on a Tescan GAIA 3 FIB/SEM microscope. For the spectra acquisition, both 15 and 20 kV modes were used in order to assess changes in the elemental concentration within the bulk of steel_168h, copper_168h, and brass_168h. The values of the concentrations result from an average among 5 different spots. A tiny amount of each sample was placed on a stub with SEM conductive adhesive on top. EDX acquisition was performed on relatively thick (>200 μm) sample portions to minimize possible contributions to C, N, and O signals from the adhesive, which is constituted by these elements. For the quantification, we used the Kα lines of the metals. EDX acquisitions were performed under low magnification in order to minimize inhomogeneity effects on the quantification.

## 3. Results and Discussion

### 3.1. Sample Appearance after Thermal Treatment

**Figure 1:**
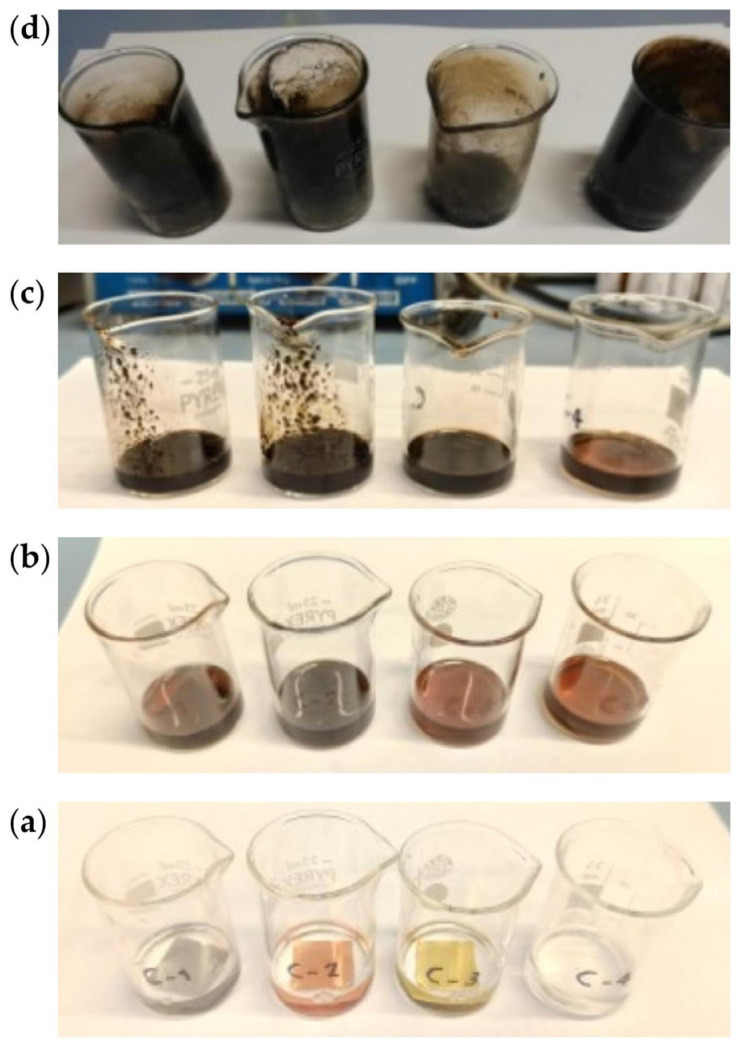
Samples of [BmPyrr]FAP after heating at 200 °C in the presence of steel, copper, and brass metal plates, and with no metal plate (from left to right) for 0 (**a**), 4 (**b**), 24 (**c**), and 168 (**d**) hours.

Figure 1 shows the appearance of the original sample and of the samples heated for 4, 24, and 168 h in the presence of steel, copper, or brass plates, or in the absence of any plate. All the samples displayed a brown color after heating for 4 h, indicating that the thermal treatment degrades [BmPyrr]FAP (Figure 1b). The color was more intense in the samples heated in the presence of the plates, particularly steel and copper, and became darker at longer heating times. After heating for 24 h (Figure 1c), a dark solid appears in the liquid heated in the presence of steel and copper. In these cases, only a spongy solid is left after 168 h, whereas in the sample containing brass and in the one with no metals, a highly viscous liquid can be observed (Figure 1d).

### 3.2. Characterization of the Thermal Degradation of the Cation

The thermal stability of the [BmPyrr] cation in the presence of steel, copper, or brass was investigated by ^1^H and ^13^C NMR and compared to that in the absence of any metal/alloy. The complete assignment of the cation ^1^H and ^13^C signals was obtained thanks to the acquisition of ^1^H and ^13^C one-dimensional spectra and of ^1^H-^1^H TOCSY and ^1^H-^13^C HSQC two-dimensional experiments on the non-heated IL, i.e., blank_0h. The assignment is reported in Table S1.

#### 3.2.1. Thermal Degradation of the Cation in the Absence of the Metal/Alloy

**Figure 2:**
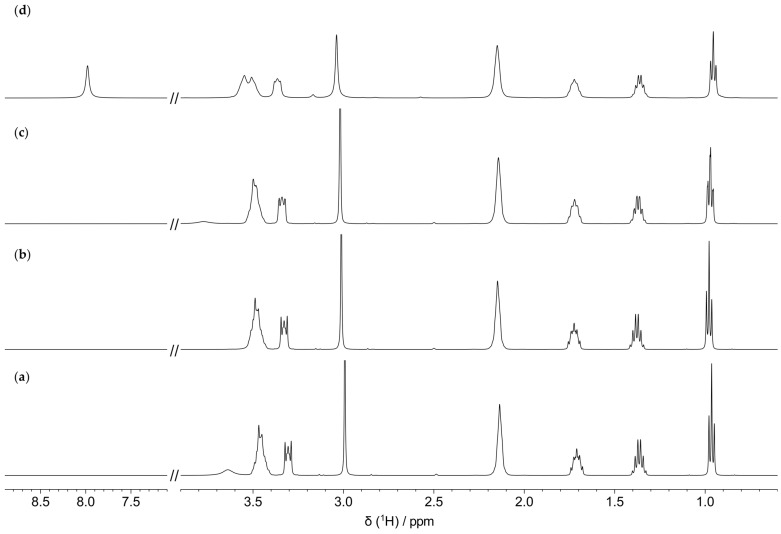
^1^H HRMAS NMR spectra of blank samples after 0 (**a**), 4 (**b**), 24 (**c**), and 168 (**d**) hours of heating.

**Figure 3:**
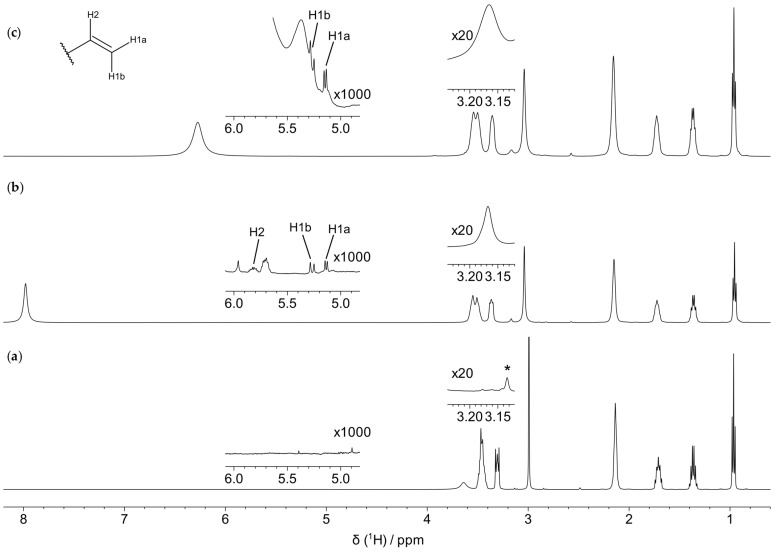
^1^H HRMAS NMR spectrum of blank_0h (**a**) compared to that of blank_168h (**b**) and of brass_168h (**c**). Signal assignments refer to hydrogen nuclei of a terminal vinyl group, as shown in the structure reported in the inset. The asterisk (*) refers to the satellite methyl signal (N-CH_3_) of the original cation.

Figure 2 shows the ^1^H HRMAS NMR spectra of the IL collected after different heating times in the absence of any metal/alloy. The main signals remain unaltered, although a slight broadening is observable upon increasing the heating time, probably due to the increase in viscosity. This piece of evidence is in agreement with the work of Salgado et al., showing that the thermal degradation of [BmPyrr]FAP for more than 5 h at 200 °C is hardly appreciable [27]. In the sample blank_168h, an intense peak resonating at about 8 ppm occurs, belonging to protons that do not show any direct correlation with ^13^C nuclei, as highlighted in the ^1^H-^13^C HSQC spectrum (Figure S1). This signal might arise from water protons rapidly exchanging with labile protons of the degradation products of the anion presented in the following section. Low-intensity peaks, due to degradation products of the cation, progressively appear upon heating and can be detected if the vertical scale is magnified, as shown in the insets of Figure 3b for the sample heated for 168 h. Specifically, a peak appears at about 3.2 ppm due to a proton in an isolated spin system. This is evidenced by the selective ^1^H TOCSY spectrum displayed in Figure S2b. This signal is also present in the ^1^H-^13^C HSQC spectrum (Figure S1) and correlates with a ^13^C signal at 51.5 ppm. Thus, we inferred that the ^1^H and ^13^C signals may arise from a methyl group linked to a heteronucleus (X-CH_3_, with X = O, N, P). By further expanding the intensity scale of the ^1^H spectrum of blank_168h, it is possible to identify two doublets and one multiplet in the region between 5 ppm and 6 ppm; these three signals are present in the ^1^H TOCSY spectrum recorded by irradiating the multiplet at 5.81 ppm (Figure S2b), indicating that they arise from protons belonging to the same spin system. Based on the correlation signals detected in the ^1^H-^13^C HSQC spectrum of blank_168h (Table S2), we assigned them to ^1^H and ^13^C nuclei of a terminal vinyl group, probably belonging to a product of the Hoffman elimination of [BmPyrr], either involving the elimination of the butyl chain, leading to 1-butene, or the opening of the pyrrole ring [22,28]. The degradation product derived from the opening of the ring was already observed for [BmPyrr] and reported in Refs. [15,29].

#### 3.2.2. Thermal Degradation of the Cation in the Presence of the Metal/Alloy

The presence of the brass metal plate during heating does not seem to promote further degradation of the IL cation; in fact, ^1^H spectrum of brass_168h is quite similar to the one recorded for blank_168h (Figure 3). In particular, in this case it is possible to detect a high intensity peak at about 6.3 ppm, which is not present in the ^1^H-^13^C HSQC spectrum, similarly to the one resonating at ~8 ppm in the ^1^H spectrum of blank_168h. Also in this case, it is probably imputable to water protons exchanging with protons of the anion degradation products. Furthermore, in this spectrum, the low intensity peak at ~3.2 ppm, assigned to the X-CH_3_ product, and the barely detectable doublets resonating between 5.0 and 5.4 ppm, due to products of the Hoffmann elimination, are present also here and are characterized by intensities comparable to those observed for blank_168h. Therefore, the presence of brass does not seem to modify the thermal degradation pathways of the IL.

In the case of the IL heated in the presence of steel, the detailed analysis of the degradation products possibly formed after heating for more than 4 h was hampered by the significant line broadening detected on the ^1^H NMR spectra of steel_24h and steel_168h (Figure S3). This broadening could be ascribed to the presence of paramagnetic species derived from the partial dissolution of the metal plate, as detected by ICP-OES (see Section 3.4). However, the spectrum of steel_4h does not evidence the presence of degradation products, as observed for blank_4h (Figure S3). This indicates a similar behavior up to 4 h of heating.

On the contrary, the ^1^H spectra recorded for the IL heated with copper evidences the presence of degradation products already after 4 h of heating, even if the signals of the cation still dominate the spectrum for up to 24 h (Figure S4b,c). Interestingly, almost complete degradation of the cation can be observed after 168 h. In fact, the signals of the cation are barely detectable, whereas broad signals show up in the region between 5 and 6 ppm. Moreover, a signal appears at ~7.3 ppm, showing a triplet structure characterized by a *J* of about 50 Hz, which was ascribed to NH_4_^+^. This signal is diagnostic of the elimination of all substituents from the [BmPyrr] cation. It is worth noting that, as revealed by ICP-OES (see Section 3.4), the amount of copper detected in copper_168h is almost three times that detected in brass_168h, where the degradation is modest; this result suggests that the extensive degradation of the cation is promoted by a larger amount of copper.

To sum up, our measurements highlight a significant stability of the cation upon thermal treatment both in the absence and in the presence of all the metal plates up to 24 h; however, copper induces a considerable degradation after 168 h of thermal treatment.

### 3.3. Characterization of the Thermal Degradation of the Anion

**Scheme 2:**
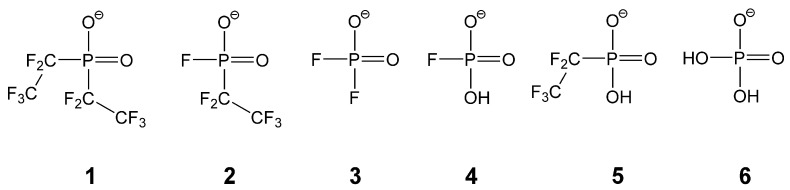
Proposed degradation products of FAP.

**Figure 4:**
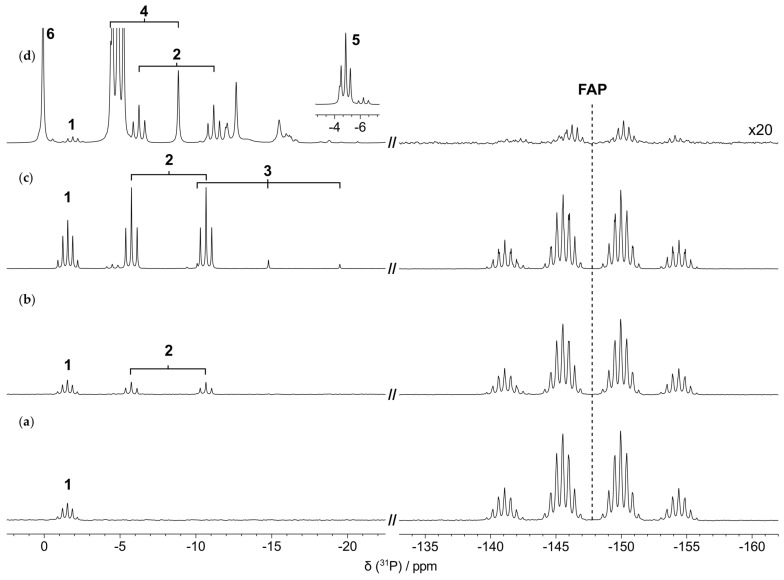
^31^P HRMAS spectra of [BmPyrr]FAP (**a**), blank_4h (**b**), blank_24h (**c**), and blank_168h (**d**). The numbers 1–6 associated to the signals refer to the degradation products pictured in Scheme 2. In trace (**d**), the portion of the spectrum ranging from −162 to −133 ppm is magnified 20 times to evidence the signal of FAP. The signal due to compound **5**, whose intensity is out of scale in trace (**d**), is shown in the inset scaled in intensity so that it is completely visible.

**Figure 5:**
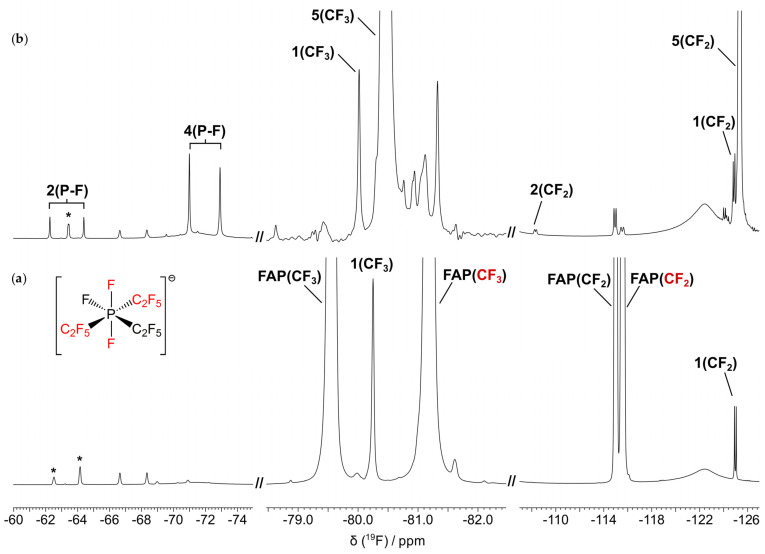
Expansions of the ^19^F spectra of [BmPyrr]FAP (**a**) and [BmPyrr]FAP heated for 168 h at 200 °C with no metal plate (blank_168h) (**b**). The non-equivalent substituents of FAP are differentiated by the black and red colours. The assignment reported on each signal refers to the degradation products 1–5 pictured in Scheme 2 and to their specific fluorine atoms (reported in brackets). The asterisks (*) mark the spinning sidebands.

^31^P and ^19^F NMR spectra were acquired for the characterization of the degradation of the FAP anion upon heating in the absence or in the presence of the metal/alloy plates. The ^31^P and ^19^F spectra of blank_0h are shown in Figure 4a and Figure S5a, respectively. They are consistent with those already reported in the literature for an IL containing FAP [30]; the ^19^F spectrum highlights the presence of *mer*-FAP-type structure, consisting of spin sets displaying different chemical shifts, assigned to: (i) two chemically and magnetically equivalent fluorine atoms; (ii) the remaining fluorine atom; (iii) two chemically and magnetically equivalent pentafluoroethyl substituents; and (iv) the remaining pentafluoroethyl substituent. The complete ^19^F assignment is reported in Table S3. The ^31^P spectrum presents a main signal at −147.7 ppm with a complex multiplicity, due to 1-bond and 2-bond *J*_P–F_ couplings between the phosphorous nucleus and the directly bound fluorine (P–F) and CF_2_, respectively. Furthermore, a second signal was detected in the region of phosphinic acids/phosphinates at −1.5 ppm, with a pentet multiplicity (*J* = 66 Hz) and an intensity of about 3% relative to the main peak (Figure 4a); also in the ^19^F spectrum, some low intensity peaks are detectable at −80.2 ppm (singlet) and at −125.0 ppm (doublet, *J* = 66 Hz) (Figure 5a). Based on their resonance frequencies and *J* coupling, these signals were assigned to CF_3_ and CF_2_ of bis(pentafluoroethyl)phosphinate (compound **1** of Scheme 2), respectively, in agreement with the literature [31]. This compound is known to be a hydrolysis product of the neutral precursor of FAP, tris(pentafluoroethyl)difluorophosphorane [32]. These data highlight that FAP undergoes a moderate degradation due to hydrolytic reactions even at room temperature.

#### 3.3.1. Thermal Degradation of the Anion in the Absence of the Metal/Alloy

**Figure 6:**
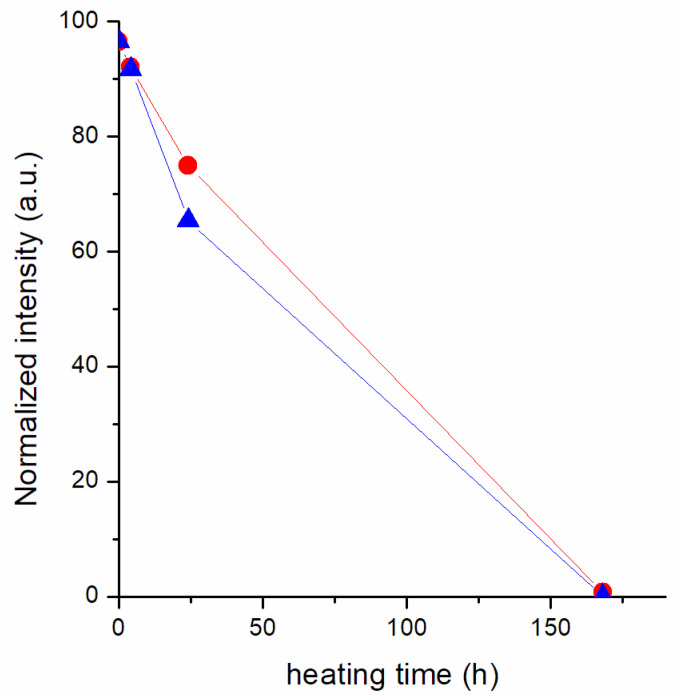
Intensity of FAP signal as a function of heating time for the samples with no metal/alloy plates (red circles, samples blank_0h, blank_4h, blank_24h, and blank_168h) and with steel (blue triangles, samples steel_0h, steel_4h, steel_24h, and steel_168h) as obtained from the ^31^P NMR spectra. The intensity is reported as the percentage of the total integral of each spectrum. The lines are added to guide the eye.

Interestingly, the ^31^P spectra of all samples collected during thermal treatment without the metal plates present new signals in the region of phosphates/phosphinates, between 5 and −25 ppm, which tend to evolve with heating time (Figure 4). Concomitantly, the main signal at −147.7 ppm progressively decreases in intensity and becomes barely detectable after 168 h of heating (Figure 4d). The decrease in intensity of the FAP signal as a function of time is reported in Figure 6, indicating a low stability of the anion to a prolonged thermal treatment, even in the absence of the metal/alloy plates. The almost complete degradation of the anion is also confirmed by the comparison of the ^19^F spectra of original IL and of the IL heated for 168 h (Figure S5 and Figure 5), the latter presenting new peaks almost completely substituting the FAP signals.

In order to identify the possible degradation products formed during heating, ^31^P spectra (Figure 4) were analyzed together with the ^19^F spectrum of blank_168h (Figure 5b, Figure S5b). Compared to the ^31^P spectrum of the original IL, the spectrum of blank_4h (Figure 4b) presents one additional signal (doublet of triplets) at −8.2 ppm, which is assignable to a compound with a P atom linked to a fluorine atom and a pentafluoroethyl group; it was hypothesized that this signal belongs to pentafluoroethylfluorophosphinate, named compound **2** in Scheme 2. After 24 h of heating, the intensity of the signals of both compounds **1** and **2** in the ^31^P spectrum increases (Figure 4c); moreover, a new triplet with *J* = 952 Hz is detectable at −14.8 ppm, belonging to a species containing a phosphorous atom directly bound to two fluorine atoms; this signal is probably due to difluorophosphate (compound **3** of Scheme 2). In the spectrum of blank_168h (Figure 4d), the signal of compound **3** is no more detectable, whereas signals of compounds **1** and **2** persist, but have a lower intensity compared to that detected in the previous spectrum; moreover, several new signals can be observed, the more intense being a triplet at −4.9 ppm (*J* = 73 Hz); the same *J* coupling was found for the signal resonating at −125.3 ppm on the ^19^F spectrum, and the two signals were assigned to pentafluoroethylphosphinate (compound **5** of Scheme 2), according with Mahmood et al. [31]. Furthermore, a doublet at −6.6 ppm (*J* = 902 Hz) was detected in the ^31^P spectrum, which shares the same *J* coupling with the doublet resonating at −71.9 ppm in the ^19^F spectrum, both signals being ascribable to monofluorophosphate (compound **4** of Scheme 2). A singlet at 0.1 ppm was observed in the ^31^P spectrum and assigned to phosphate (compound **6** of Scheme 2). Finally, a singlet centered at ~−148 ppm is present in the ^19^F spectrum, probably due to F^−^. The complete set of ^31^P and ^19^F chemical shifts and *J* couplings of all the degradation compounds is reported in Table 2 and Table 3. It should be noticed that our data do not allow us to determine the protonation state of the degradation compounds. All the compounds identified indicate that FAP underwent hydrolysis, but it is not possible to state whether hydrolysis occurred during heating or when the samples were unloaded from the oven. Anyway, ^19^F and ^31^P NMR data highlight that FAP undergoes severe degradation upon heating at 200 °C for 168 h even in the absence of any plate.

#### 3.3.2. Thermal Degradation of the Anion in the Presence of the Metal/Alloy

**Figure 7:**
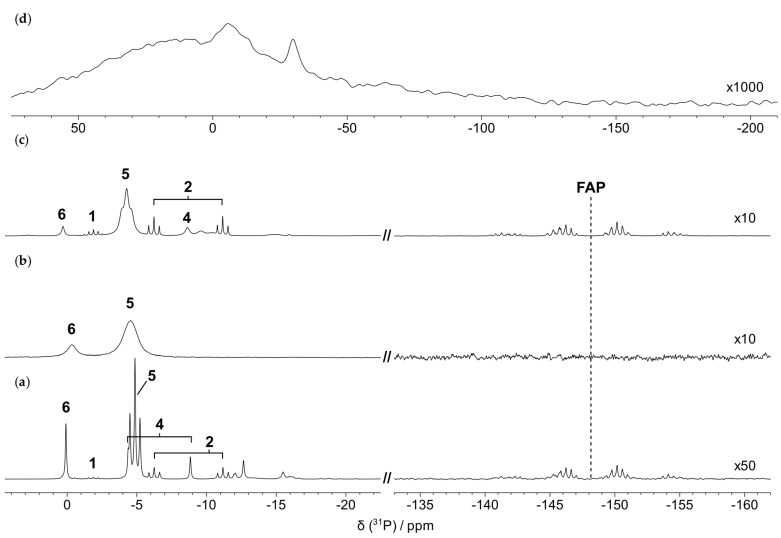
^31^P HRMAS spectra of blank_168h (**a**), steel_168h (**b**), brass_168h (**c**), and copper_168h (**d**). In traces (**a**–**c**), the portion of the spectra ranging from −162 to −133 ppm is magnified as reported in the figure. The assignment reported on each signal refers to the degradation products 1–6 pictured in Scheme 2.

^31^P spectra were also recorded for the IL heated for 168 h in the presence of the metal plates. These spectra are shown in Figure 7 in comparison with the one recorded for blank_168h. In the case of steel_168h, the main signal of FAP is no more detectable due to complete degradation; on the other hand, two broad signals appear at −0.3 and −4.5 ppm, probably mainly due to compound **5** and compound **6** of Scheme 2, already observed at similar frequencies in the spectrum of blank_168h. Here, the signals are quite broad because of the presence of paramagnetic iron species in the sample, as detected by ICP-OES and EDX. In the case of brass_168h, the remaining FAP ion accounts for only about 3% of the total ^31^P spectrum integral, with degradation products appearing in the region between 1 and −17 ppm. Interestingly, signals ascribable to compounds **1** and **2** are quite narrow, while signals assigned to compounds **4**, **5**, and **6** are significantly broader; this result suggests that the last three species are in close proximity and might coordinate paramagnetic species dissolved in the sample, such as Cu(II). The ^31^P spectrum of copper_168h shows extremely broad signals in the high frequency spectral region, above ~−50 ppm, probably due to the presence of a high amount of paramagnetic Cu(II), and the signal of FAP is no longer observable. This suggests a complete degradation of FAP also for this sample.

Therefore, despite the fact that FAP has a higher stability to hydrolysis compared to the [PF_6_]^−^ anion, our data clearly show that it is not suitable for applications involving heating at high temperatures for prolonged times.

### 3.4. Detection of Elements in the Heated IL Using ICP-OES and EDX

The metals present in the plates were searched in the ionic liquid after thermal treatment by ICP-OES. In particular, in the case of the IL heated in the presence of the steel plate, Fe, Ni, and Cr were searched, and for the one heated with the copper plate, Cu was searched, whereas for the IL heated with the brass plate, Cu, Zn, and Sn were searched. In the ionic liquid heated in the absence of any metal plate, Fe, Ni, Cr, Cu, Sn, and Zn were searched and the metal contents were under detection limits for all heating times.

**Figure 8:**
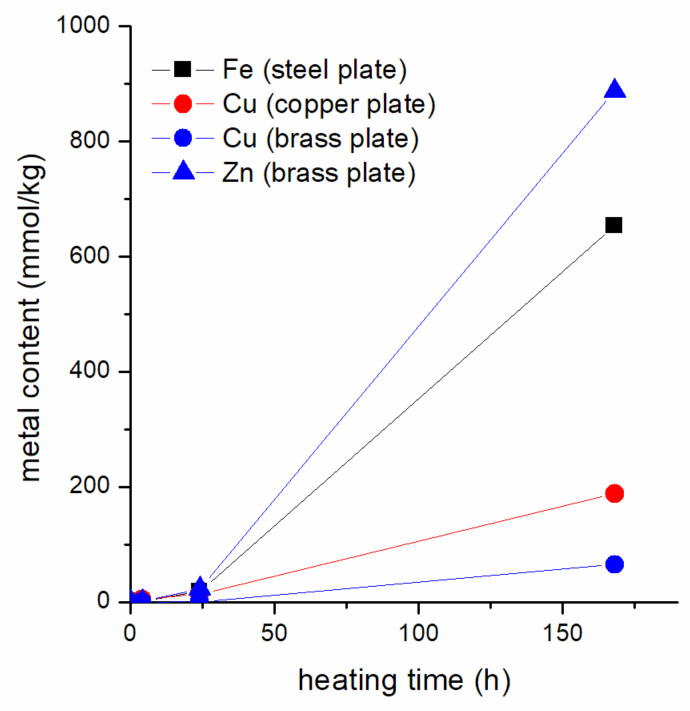
Metal content in the ionic liquid heated in the presence of the steel (black), copper (red), or brass (blue) plates as a function of the heating time as detected by ICP-OES. The symbols indicate the detected metal (square for Fe, circle for Cu, and triangle for Zn).

The metal contents of the samples heated in the presence of the metal plates at different heating times are reported in Table 4 and shown in Figure 8. Ni and Sn contents were under the detection limit in all samples. The data show that the metal content increases upon increasing the heating time. Fe and Zn contents in the ionic liquid heated for 168 h in the presence of steel and brass, respectively, are significantly higher than the copper amount detected in the case of the IL heated with the copper plate. Likewise, a smaller amount of copper than zinc was detected from the brass plate samples, demonstrating a lower tendency of Cu to be leached into the ionic liquid during heating with respect to Zn.

The EDX technique was used to detect elements associated with the IL (C, N, O, F, and P) and with the metal/alloy plate within the bulk of steel_168h, copper_168h, and brass_168h samples. Table 5 summarizes the average atomic percentages of each identified element for each sample. For steel_168h, EDX detected Fe as a “dissolved” element from the metal plate and Cu in copper_168h. The data collected from the ionic liquid containing the brass piece (brass_168h) showed only the presence of Zn dissolved and no Cu. The amounts of detected metals are consistent with those revealed by ICP-OES. In the case of brass_168h, Cu was not detected by EDX, probably due to a higher detection limit with respect to ICP-OES.

By comparing the atomic percentages calculated for the pure IL (Table 5, column headed “[BmPyrr]FAP”) and the ones of the analyzed samples (Table 5, columns headed “Steel_168h”, “Copper_168h”, and “Brass_168h”), a significant decrease in fluorine amount and a strong increase in oxygen can be noticed on the analyzed samples. Furthermore, an increase in phosphorous content is detectable. These trends can be rationalized considering the degradation products identified by NMR. Indeed, the atomic percentages calculated for the ionic pairs composed by [BmPyrr] and FAP main degradation products (i.e. compounds **5** and **6**, Table 5, columns headed “[BmPyrr][Compound **5**]” and “[BmPyrr][Compound **6**]”) show that, in the degradation products, O and P increase compared to [BmPyrr]FAP, whereas F decreases. Therefore, EDX data are in line with the formation of the FAP degradation products revealed by NMR and discussed in Section 3.3. Since fluorine content decreases in the degraded samples, we can hypothesize the formation of volatile fluorine species, even if no evidence supporting this hypothesis was found in the literature, as the identification of FAP degradation products is still a rather unexplored subject.

## 4. Conclusions

In this work, we characterized the thermal degradation of [BmPyrr]FAP, which is a potential IL to be used for TES in solar energy applications. The IL was subjected to thermal treatment at 200 °C and monitored after 4, 24, and 168 h of heating. The effect of three different metal/alloys, i.e., steel, copper, and brass, on the thermal degradation of [BmPyrr]FAP was also studied. The conditions tested are relevant in solar-concentrating power plants. HRMAS NMR allowed us to characterize the degradation products of both the cation and the anion, while ICP-OES provided the content of metals dissolved in the ionic liquid and EDX was used for quantifying O and F content in the degraded IL. The NMR characterization showed that the cation is quite stable alone and against all the metals/alloys up to 24 h of heating. After 168 h, it is considerably degraded when heated in contact with copper, while it remains practically unchanged in the case of brass. Unfortunately, severe line broadening due to dissolved iron prevented us from ascertaining the cation stability against steel. On the other hand, the FAP anion completely degrades even in the absence of any metal/alloy, progressively undergoing hydrolysis reactions. FAP hydrolysis is quite noticeable considering the low amount of water dissolved in [BmPyrr]FAP in normal conditions. Steel and brass do not seem to sensibly affect the anion degradation. In fact, the main FAP degradation products are pentafluoroethylphosphinate and phosphate, as observed in the case of the IL degraded in the absence of metal/alloy. In all cases, these products are also present in similar relative amounts. Overall, our analysis highlights that [BmPyrr]FAP is not suitable to be employed for prolonged thermal exposure, even with no contact with metal/alloy plates, mainly due to the FAP anion. However, the [BmPyrr] cation shows a substantial thermal stability also when in contact with steel and brass, and can be considered a promising cation for the design of novel ILs to be used as working fluids for heat storage applications.

## Figures and Tables

**Table 1:** Codes of the samples.

Metal/Alloy	4 h at 200 °C	24 h at 200 °C	168 h at 200 °C
steel	steel_4h	steel_24h	steel_168h
copper	copper_4h	copper_24h	copper_168h
brass	brass_4h	brass_24h	brass_168h
-	blank_4h	blank_24h	blank_168h

**Table 2:** ^31^P NMR chemical shifts and *J*_P–F_ couplings assigned to the degradation compounds **1**–**6** pictured in Scheme 2. Signal multiplicity is reported in brackets.

Species	^31^P δ (ppm) ^a^	*J*_P–F_ (Hz)
**1**	−1.9 (p)	66 (2 bonds)
**2**	−8.7 (dt)	998 (1 bond), 74 (2 bonds)
**3**	−14.8 (t)	951 (1 bond)
**4**	−6.6 (d)	902 (1 bond)
**5**	−4.9 (t)	73 (2 bonds)
**6**	0.1 (s)	-

^a^ Abbreviations: s = singlet, d = doublet, t = triplet, and p = pentet.

**Table 3:** ^19^F NMR chemical shifts and *J*_P–F_ couplings assigned to specific fluorine nuclei of the degradation compounds **1**–**5** pictured in Scheme 2 and to the fluoride anion. Signal multiplicity is reported in brackets.

Species	^19^F δ (ppm) ^a^	*J*_P–F_ (Hz)
**1** (CF_2_)	−124.9 (d)	66 (2 bonds)
**1** (CF_3_)	−80.0 (s)	-
**2** (P–F)	−63.3 (d)	998 (1 bond)
**2** (CF_2_)	−108.3 (d)	74 (2 bonds)
**4** (P–F)	−71.9 (d)	902 (1 bond)
**5** (CF_2_)	−125.3 (d)	73 (2 bonds)
**5** (CF_3_)	−80.5 (s)	-
**F^−^**	−148.2 (s)	-

^a^ Abbreviations: s = singlet, d = doublet.

**Table 4:** Metal content (mg kg^−1^) in the ionic liquid heated in the presence of the steel, copper, or brass plates at different heating times by ICP-OES.

Sample	Fe	Cr	Cu	Zn
steel_4h	97.2	n.d.		
steel_24h	1033	n.d.		
steel_168h	36,500	11.2		
copper_4h			304	
copper_24h			896	
copper_168h			11,954	
brass_4h			23.2	70.5
brass_24h			57.7	1510
brass_168h			4146	58,021

**Table 5:** Atomic percentages of the ionic liquid after 168 h of thermal treatment in the presence of steel, copper, and brass metal plates, as determined by EDX elemental analysis. The atomic percentages calculated for pure IL, [BmPyrr][Compound **5**] and [BmPyrr][Compound **6**] are also displayed for comparison.

Element	Atomic%					
	Steel_168h	Copper_168h	Brass_168h	[BmPyrr]FAP ^a^	[BmPyrr] [Compound **5**] ^a^	[BmPyrr] [Compound **6**] ^a^
C	40.2	38.8	43.5	43	52	60
N	6.7	8.3	4.8	3	5	7
O	28.4	30.8	21.3	0	14	27
F	19.7	16.9	24.7	51	24	/
P	4.2	4.7	4.8	3	5	7
Fe	0.8 (32) ^b^	/	/	/	/	/
Cu	/	0.4 (16) ^b^	/	/	/	/
Zn	/	/	0.9 (39) ^b^	/	/	/
Total	100	100	100	100	100	100

^a^ Calculated from the chemical formulas of [BmPyrr]FAP (C_11_NO_3_F_5_P), [BmPyrr]compound **5** (C_11_NO_3_F_5_P) and [BmPyrr]compound **6** (C_9_NO_4_P) without hydrogen atoms. ^b^ The number in brackets indicates the metal content expressed in mg g^−1^.

## Data Availability

The data presented in this study are available within the article or supplementary material and on request from the corresponding authors.

## Supplementary Materials

The following supporting information can be downloaded at: https://www.mdpi.com/article/10.3390/ma16051762/s1, Scheme S1: Numbering of the hydrogen atoms of [BmPyrr] used in the ^1^H signal assignment reported in Table S1; Figure S1: ^1^H-^13^C HSQC spectrum of blank_168h; Figure S2: Comparison of ^1^H spectrum of blank_168h (a) with 1D selective ^1^H TOCSY obtained by irradiating the methyl protons of X-CH_3_ at 3.17 ppm (b). Comparison of the ^1^H spectral region ranging between about 4.5 and 6.0 ppm of blank_168h (c) with 1D selective ^1^H TOCSY spectra obtained by irradiating H2 nucleus at 5.81 ppm (d) and H1b nucleus at 5.14 ppm (e) carried by the terminal vinyl group pictured in the inset; Figure S3: ^1^H HRMAS NMR spectra of blank_0h (a), steel _4h (b), steel_24h (c) and steel_168h (d); Figure S4: ^1^H HRMAS NMR spectra of blank_0h (a), copper _4h (b), copper_24h (c) and copper_168h (d). Residual water and DMSO are marked with asterisks; Figure S5: ^19^F spectra of blank_0h (a) and blank_168h (b); Table S1: Assignment of ^1^H and ^13^C NMR signals of the [BmPyrr] cation according to the numbering reported in Scheme S1. Signal multiplicity and integration are reported in brackets; Table S2: Correlations between ^1^H and ^13^C nuclei of the degradation products, as detected from the ^1^H-^13^C HSQC spectrum. Signal multiplicity and integration are reported in brackets; Table S3: Assignment of ^19^F NMR signals and ^19^F-^31^P *J* couplings (*J*_P–F_) of the FAP anion of the investigated ionic liquid. The different colors are referred to fluorine atoms in different environments, as reported in the structure of Figure S5. Signal multiplicity and integration are reported in brackets.

## References

[1] Hauer A. (2022). Advances in Energy Storage: Latest Developments from R&D to the Market.

[2] Cabeza L.F. (2014). Advances in Thermal Energy Storage Systems: Methods and Applications.

[3] DeLovato N., Sundarnath K., Cvijovic L., Kota K., Kuravi S. (2019). A Review of Heat Recovery Applications for Solar and Geothermal Power Plants. Renew. Sustain. Energy Rev..

[4] Mourad A., Aissa A., Said Z., Younis O., Iqbal M., Alazzam A. (2022). Recent Advances on the Applications of Phase Change Materials for Solar Collectors, Practical Limitations, and Challenges: A Critical Review. J. Energy Storage.

[5] Khan M.I., Asfand F., Al-Ghamdi S.G. (2022). Progress in Research and Technological Advancements of Thermal Energy Storage Systems for Concentrated Solar Power. J. Energy Storage.

[6] Piper S.L., Kar M., MacFarlane D.R., Matuszek K., Pringle J.M. (2022). Ionic Liquids for Renewable Thermal Energy Storage—A Perspective. Green Chem..

[7] Welton T. (2018). Ionic Liquids: A Brief History. Biophys. Rev..

[8] Zhu J., Bai L., Chen B., Fei W. (2009). Thermodynamical Properties of Phase Change Materials Based on Ionic Liquids. Chem. Eng. J..

[9] Paul T.C., Morshed A.K.M.M., Fox E.B., Visser A.E., Bridges N.J., Khan J.A. (2014). Thermal Performance of Ionic Liquids for Solar Thermal Applications. Exp. Therm. Fluid Sci..

[10] Zhang Z., Salih A.A.M., Li M., Yang B. (2014). Synthesis and Characterization of Functionalized Ionic Liquids for Thermal Storage. Energy Fuels.

[11] Das L., Rubbi F., Habib K., Aslfattahi N., Saidur R., Baran Saha B., Algarni S., Irshad K., Alqahtani T. (2021). State-of-the-Art Ionic Liquid & Ionanofluids Incorporated with Advanced Nanomaterials for Solar Energy Applications. J. Mol. Liq..

[12] Maton C., De Vos N., Stevens C.V. (2013). Ionic Liquid Thermal Stabilities: Decomposition Mechanisms and Analysis Tools. Chem. Soc. Rev..

[13] Xu C., Cheng Z. (2021). Thermal Stability of Ionic Liquids: Current Status and Prospects for Future Development. Processes.

[14] Chen Y., Han X., Liu Z., Li Y., Sun H., Wang H., Wang J. (2022). Thermal Decomposition and Volatility of Ionic Liquids: Factors, Evaluation and Strategies. J. Mol. Liq..

[15] Preibisch Y., Horsthemke F., Winter M., Nowak S., Best A.S. (2020). Is the Cation Innocent? An Analytical Approach on the Cationic Decomposition Behavior of *N*-Butyl-*N*-Methylpyrrolidinium Bis(Trifluoromethanesulfonyl)Imide in Contact with Lithium Metal. Chem. Mater..

[16] Pyschik M., Winter M., Nowak S. (2017). Determination and Quantification of Cations in Ionic Liquids by Capillary Electrophoresis-Mass Spectrometry. J. Chromatogr. A.

[17] Pyschik M., Schultz C., Passerini S., Winter M., Nowak S. (2015). Aging of Cations of Ionic Liquids Monitored by Ion Chromatography Hyphenated to an Electrospray Ionization Mass Spectrometer. Electrochim. Acta.

[18] Ananikov V.P. (2011). Characterization of Molecular Systems and Monitoring of Chemical Reactions in Ionic Liquids by Nuclear Magnetic Resonance Spectroscopy. Chem. Rev..

[19] Damodaran K. (2022). Recent Advances in NMR Spectroscopy of Ionic Liquids. Prog. Nucl. Magn. Reson. Spectrosc..

[20] Rencurosi A., Lay L., Russo G., Prosperi D., Poletti L., Caneva E. (2007). HRMAS NMR Analysis in Neat Ionic Liquids: A Powerful Tool to Investigate Complex Organic Molecules and Monitor Chemical Reactions. Green Chem..

[21] Alam T.M., Jenkins J.E., Farrukh M.A. (2012). HR-MAS NMR Spectroscopy in Material Science. Advanced Aspects of Spectroscopy.

[22] Nardelli F., Bramanti E., Lavacchi A., Pizzanelli S., Campanella B., Forte C., Berretti E., Freni A. (2022). Thermal Stability of Ionic Liquids: Effect of Metals. Appl. Sci..

[23] Ignat’ev N.V., Welz-Biermann U., Kucheryna A., Bissky G., Willner H. (2005). New Ionic Liquids with Tris(Perfluoroalkyl)Trifluorophosphate (FAP) Anions. J. Fluor. Chem..

[24] Ignat’ev N.V., Bader J., Koppe K., Hoge B., Willner H. (2015). Recent Progress in Perfluoroalkyl-Phosphorus Chemistry. J. Fluor. Chem..

[25] Noori S., Diamanti M.V., Pedeferri M., Brenna A., Ormellese M. (2018). Effect of Water Content on the Corrosiveness of Imidazolium-Based Ionic Liquids. Mater. Corros..

[26] Perissi I., Bardi U., Caporali S., Fossati A., Lavacchi A. (2008). Ionic Liquids as Diathermic Fluids for Solar Trough Collectors’ Technology: A Corrosion Study. Sol. Energy Mater. Sol. Cells.

[27] Salgado J., Parajó J.J., Fernández J., Villanueva M. (2014). Long-Term Thermal Stability of Some 1-Butyl-1-Methylpyrrolidinium Ionic Liquids. J. Chem. Thermodyn..

[28] Sowmiah S., Srinivasadesikan V., Tseng M.C., Chu Y.H. (2009). On the Chemical Stabilities of Ionic Liquids. Molecules.

[29] Preibisch Y., Peschel C., Dohmann J.F., Winter M., Nowak S. (2021). Application of Gas Chromatography Hyphenated to Atmospheric Pressure Chemical Ionization-Quadrupole-Time-of-Flight-Mass Spectrometry (GC-APCI-Q-TOF-MS) for Structure Elucidation of Degradation Products Based on the Cation in Pyr14TFSI. J. Electrochem. Soc..

[30] Föhrenbacher S.A., Krahfuss M.J., Zapf L., Friedrich A., Ignat’ev N.V., Finze M., Radius U. (2021). Tris(Pentafluoroethyl)Difluorophosphorane: A Versatile Fluoride Acceptor for Transition Metal Chemistry. Chem.–Eur. J..

[31] Mahmood T., Shreeve J.M. (1986). New Perfluoroalkylphosphonic and Bis(Perfluoroalkyl)Phosphinic Acids and Their Precursors. Inorg. Chem..

[32] Ignat’ev N.V., Willner H., Sartori P. (2009). Electrochemical Fluorination (Simons Process)—A Powerful Tool for the Preparation of New Conducting Salts, Ionic Liquids and Strong Brønsted Acids. J. Fluor. Chem..

