# Supplementary material for: Ionic Liquids as Working Fluids for Heat Storage Applications: Decomposition Behavior of N-Butyl-N-methylpyrrolidinium tris(pentafluoroethyl)trifluorophosphate
Source: Materials (Basel). 2023 Feb 21;16(5):1762. doi: 10.3390/ma16051762 (PMC10003908; doi:10.3390/ma16051762)
Supplement: Supplementary file 1 [file materials-16-01762-s001.zip › materials-2212008-supplementary.pdf]

Article

Ionic Liquids as Working Fluids for Heat Storage Applications: Decomposition Behavior of N-Butyl-N-methylpyrrolidinium tris(pentafluoroethyl)trifluorophosphate

Francesca Nardelli [1], Enrico Berretti [2], Alessandro Lavacchi [2], Emanuela Pitzalis [1], Angelo Freni [1,*] and Silvia Pizzanelli [1,3,*]

1 Italian National Council for Research—Institute for the Chemistry of OrganoMetallic Compounds, CNR-ICCOM, Via G. Moruzzi 1, 56124 Pisa, Italy
2 Italian National Council for Research—Institute for the Chemistry of OrganoMetallic Compounds, CNR-ICCOM, Via Madonna del Piano 10, 50019 Firenze, Italy
3 Centre for Instrument Sharing (CISUP), University of Pisa, Lungarno Pacinotti 43, 56126 Pisa, Italy
* Correspondence: angelo.freni@pi.iccom.cnr.it (A.F.); silvia.pizzanelli@pi.iccom.cnr.it (S.P.)

Supplementary Material

Table S1. Assignment of ^{1}H and ^{13}C NMR signals of the [BmPyrr] cation according to the numbering reported in Scheme S1. Signal multiplicity and relative integral values are reported in brackets [a].

**Assignment**	**^{1}H δ (ppm)**	**^{13}C δ (ppm)**
2	2.99 (s, 3)	47.7 (1)
3	3.31 (m, 2)	63.8 (1)
4	1.71 (m, 2)	21.2 (1)
5	1.37 (m, 2)	19.4 (1)
6	0.96 (t, 3)	12.9 (1)
7	3.46 (m, 4)	63.9 (2)
8	2.14 (m, 4)	25.2 (2)

[a] Abbreviations: s = singlet, t = triplet, m = multiplet.

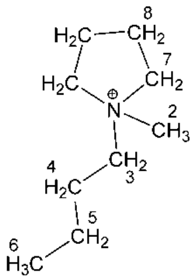

[BmPyrr]

Scheme S1. Numbering of the hydrogen atoms of [BmPyrr] used in the ^{1}H signal assignment reported in Table S1.

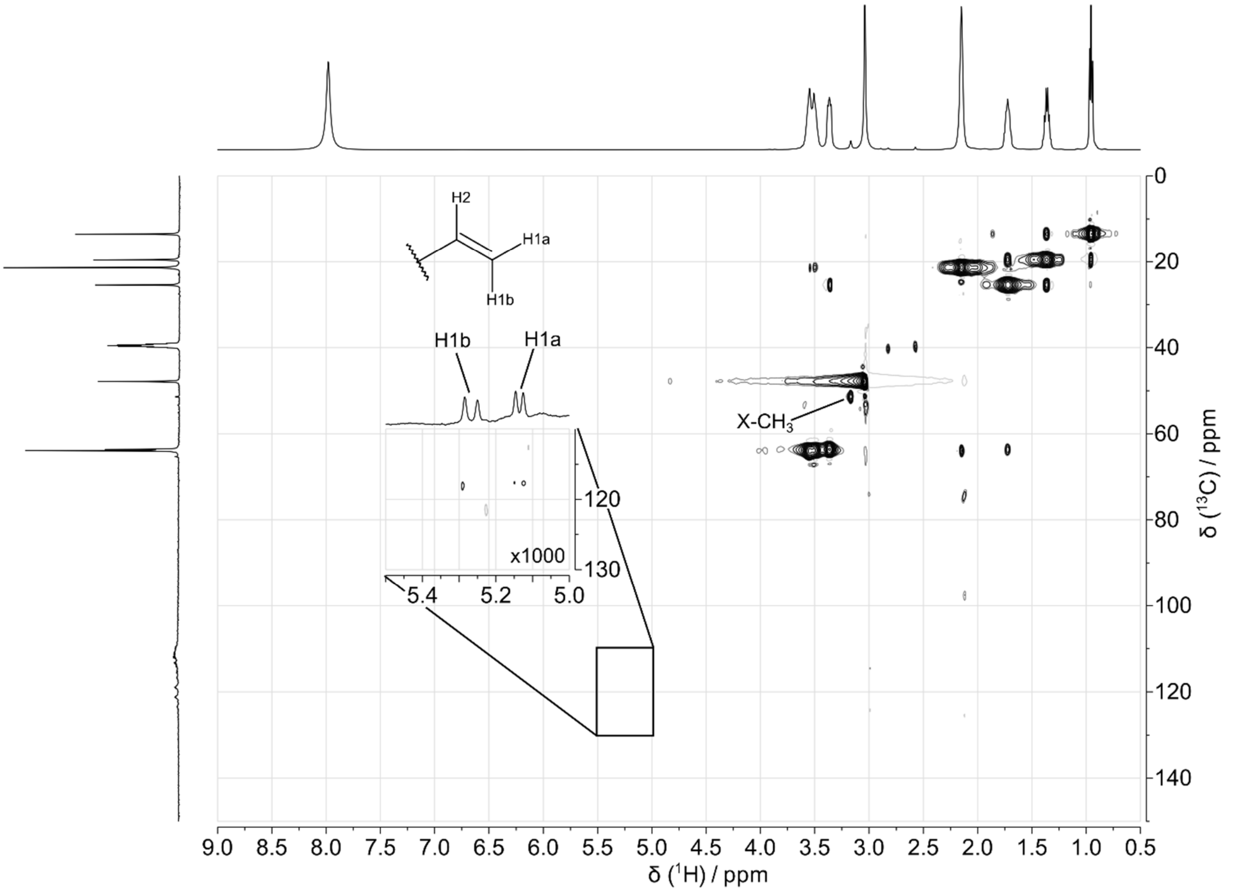

Figure S1. ^{1}H-^{13}C HSQC spectrum of blank_168h.

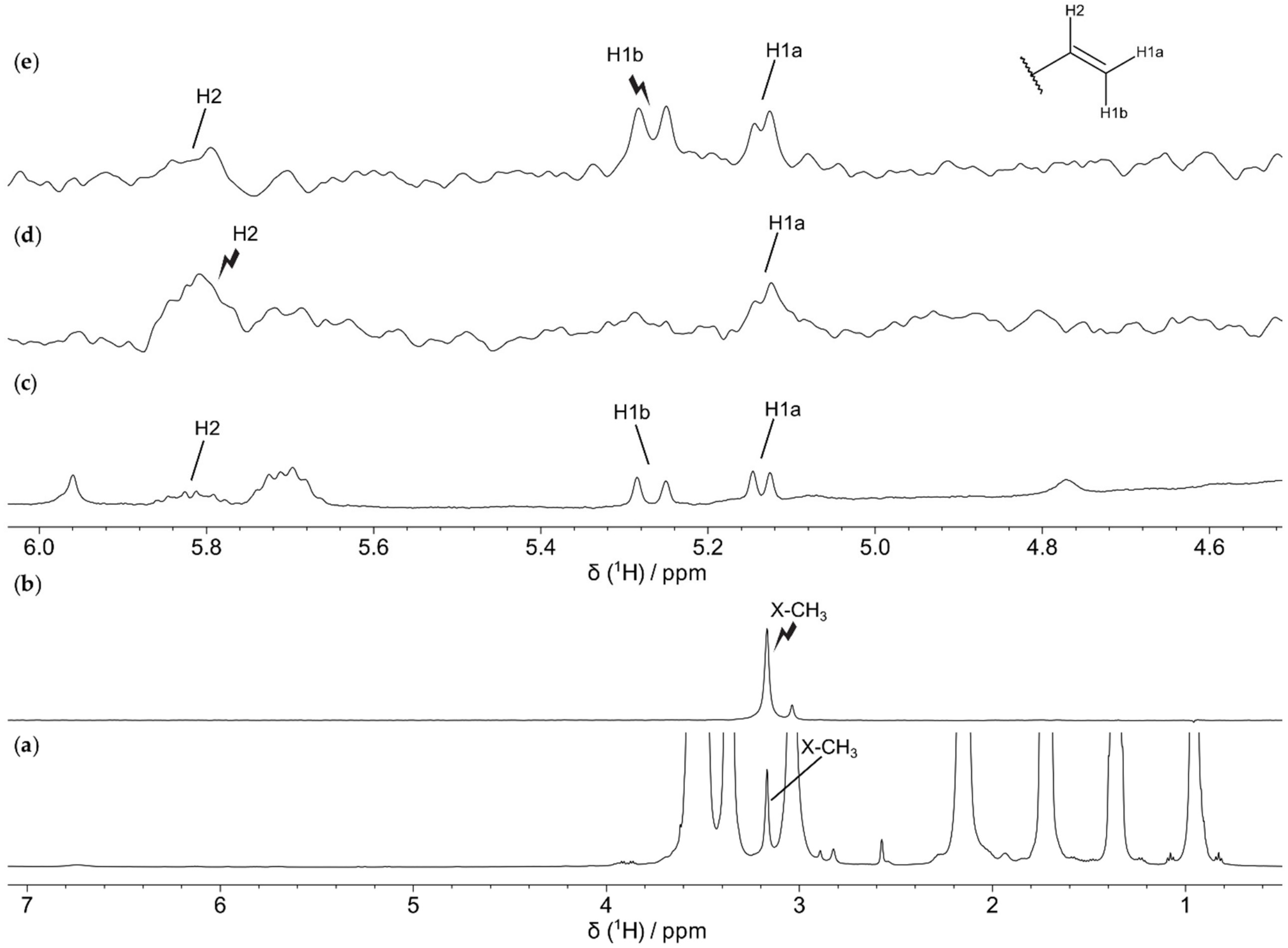

Figure S2. Comparison of ¹H spectrum of blank_168h (**a**) with 1D selective ¹H TOCSY obtained by irradiating the methyl protons of X-CH_3 at 3.17 ppm (**b**). Comparison of the ¹H spectral region ranging between about 4.5 and 6.0 ppm of blank_168h (**c**) with 1D selective ¹H TOCSY spectra obtained by irradiating H2 nucleus at 5.81 ppm (**d**) and H1b nucleus at 5.14 ppm (**e**) carried by the terminal vinyl group pictured in the inset.

Table S2. Correlations between ¹H and ¹³C nuclei of the degradation products, as detected from the ¹H-¹³C HSQC spectrum. Signal multiplicity and integration are reported in brackets [a].

¹H δ (ppm)	¹³C δ (ppm)
3.17 (s)	51.3
5.14 (d)	118.3
5.27 (d)	
5.81 (m)	-

[a] Abbreviations: s = singlet, d = doublet, m = multiplet.

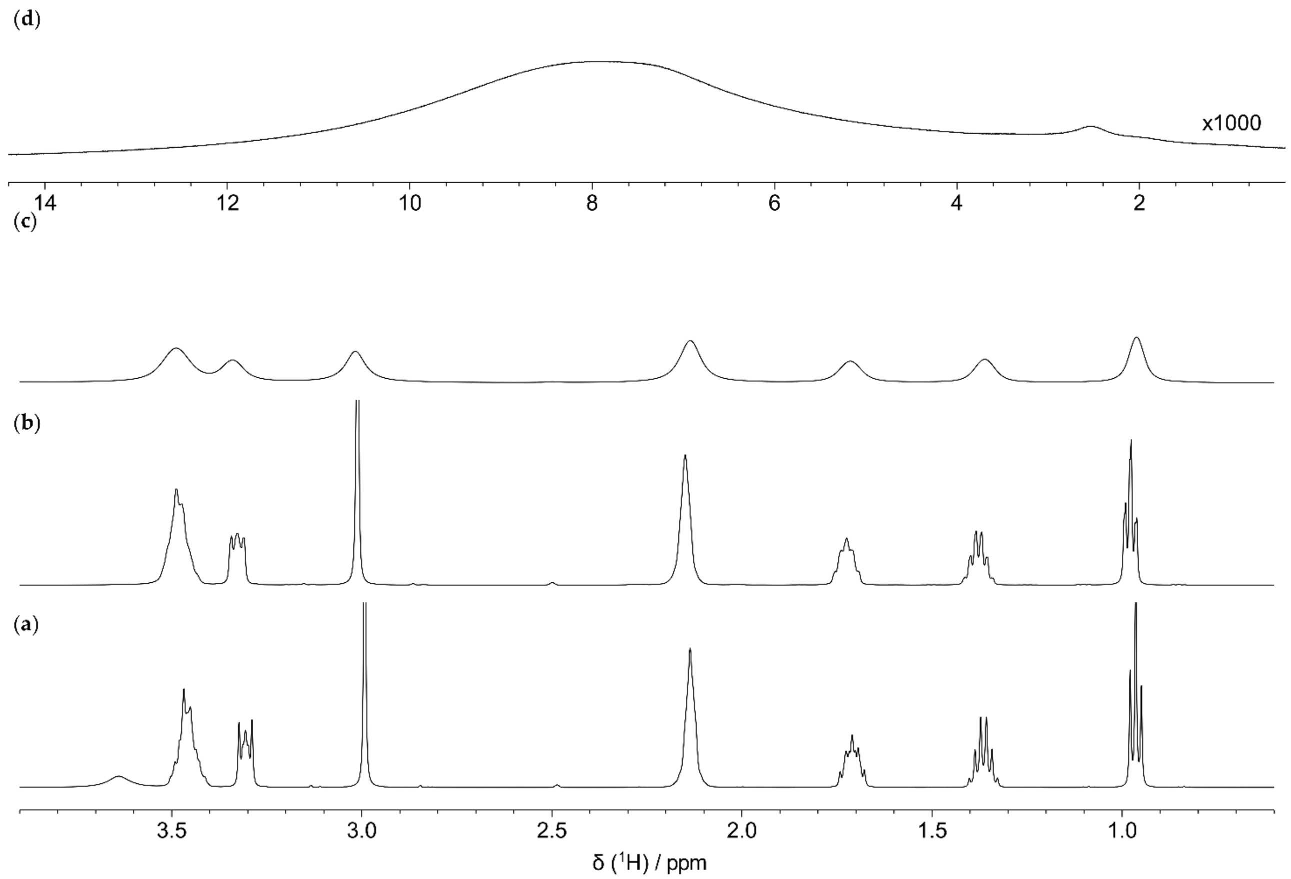

Figure S3. ^{1}H HRMAS NMR spectra of blank_0h (**a**), steel _4h (**b**), steel_24h (**c**) and steel_168h (**d**).

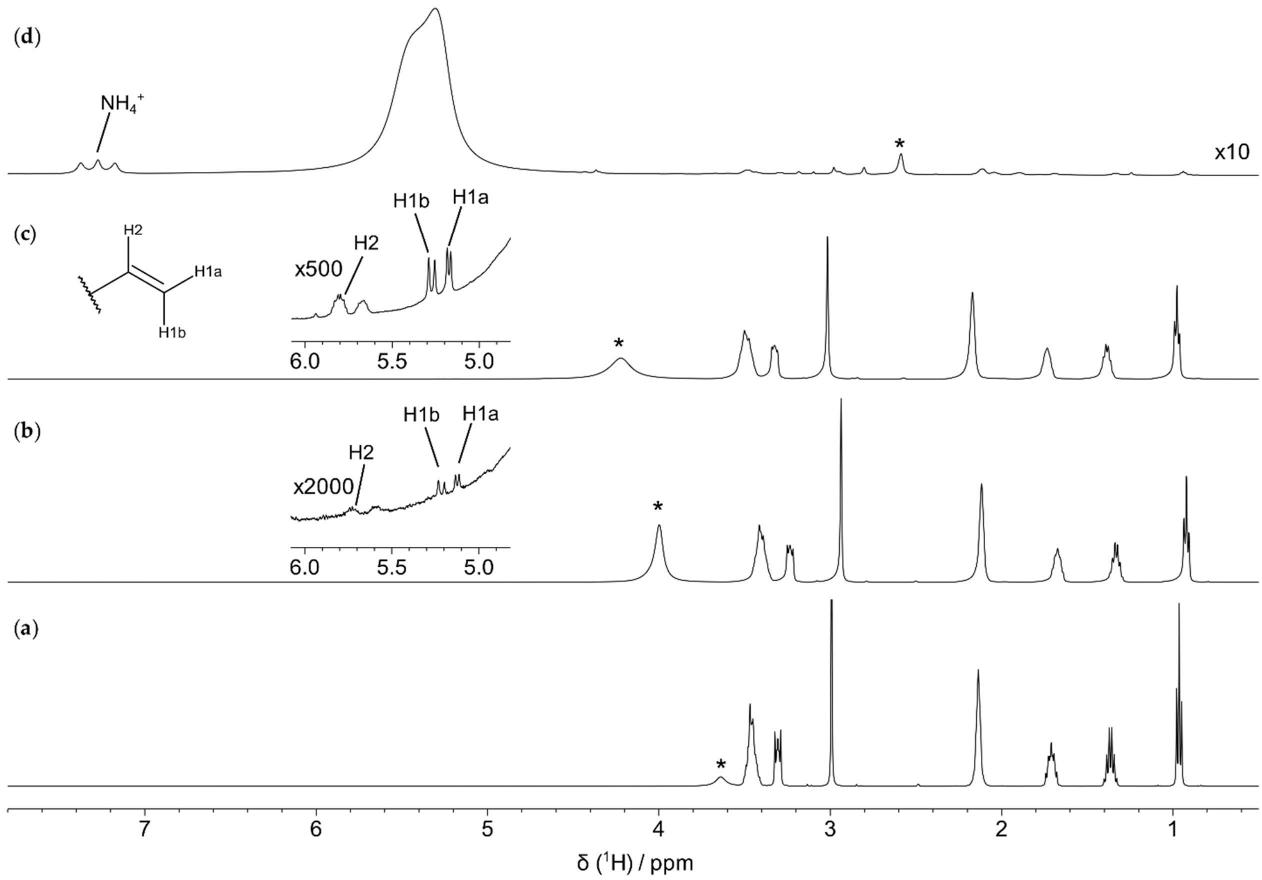

Figure S4. ^{1}H HRMAS NMR spectra of blank_0h (**a**), copper _4h (**b**), copper_24h (**c**) and copper_168h (**d**). Residual water and DMSO are marked with asterisks.

Table S3. Assignment of ^{19}F NMR signals and ^{19}F-^{31}P J couplings ($J_{P\text{-}F}$) of the FAP anion of the investigated ionic liquid. The different colors are referred to fluorine atoms in different environments, as reported in the structure of Figure S5. Signal multiplicity and integration are reported in brackets [a].

Assignment	^{19}F δ (ppm)	$J_{P\text{-}F}$ (Hz)
P-**F**	-87.0 (dm, 2)	903 (1 bond)
P-**F**	-43.7 (dm, 1)	891 (1 bond)
**CF_2**	-115.6 (dm, 4)	98 (2 bonds)
**CF_2**	-115.0 (dm, 2)	83 (2 bonds)
**CF_3**	-81.2 (m, 6)	-
**CF_3**	-79.6 (m, 3)	-

[a] Abbreviations: s = singlet, d = doublet, t = triplet.

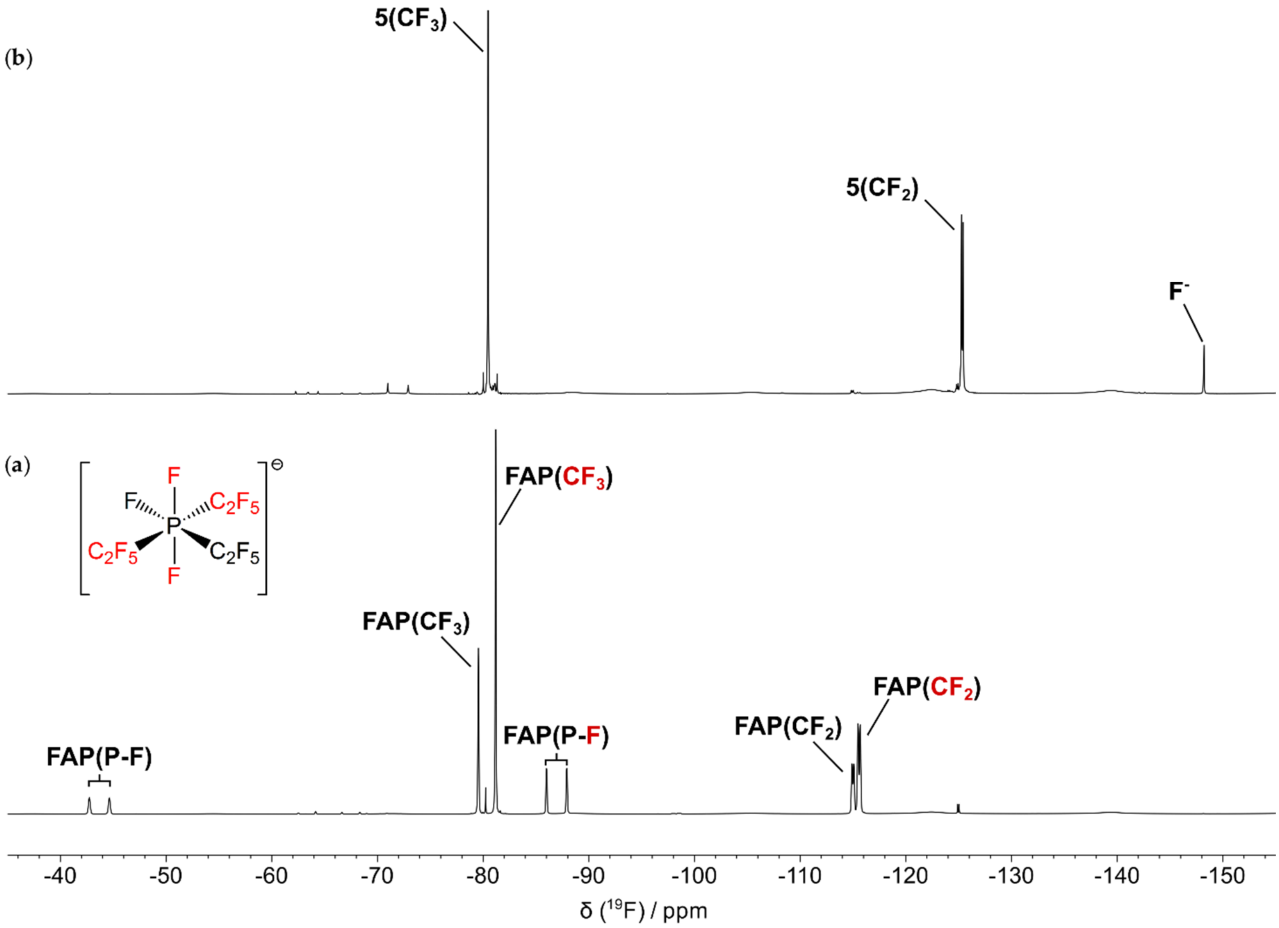

Figure S5. ^{19}F spectra of blank_0h (**a**) and blank_168h (**b**).
